# Dyslipidemia prevalence, awareness, treatment, control, and risk factors in Chinese rural population: the Henan rural cohort study

**DOI:** 10.1186/s12944-018-0768-7

**Published:** 2018-05-22

**Authors:** Xiaotian Liu, Songcheng Yu, Zhenxing Mao, Yuqian Li, Haiqing Zhang, Kaili Yang, Honglei Zhang, Ruihua Liu, Xinling Qian, Linlin Li, Ronghai Bie, Chongjian Wang

**Affiliations:** 1 Department of Epidemiology and Biostatistics, College of Public Health, Zhengzhou University, 100 Kexue Avenue, Zhengzhou, 450001 Henan People’s Republic of China; 2 Department of Nutrition and Food Hygiene, College of Public Health, Zhengzhou University, Zhengzhou, Henan People’s Republic of China; 3 Department of Clinical Pharmacology, School of Pharmaceutical Science, Zhengzhou University, Zhengzhou, Henan People’s Republic of China

**Keywords:** Dyslipidemia, Prevalence, Awareness, Treatment, Control, Risk factors, Rural

## Abstract

**Background:**

The prevalence of dyslipidemia continue to increase in recent decades in China, however, little is known about the recent prevalence, awareness, treatment, control, and potential risk factors of dyslipidemia in the rural areas of China.

**Methods:**

A total of 39,207 participants aged 18–79 years were recruited for the epidemiological research from the Henan Rural Cohort study. The age- and sex-adjusted means (95% confidence intervals, *CI*) of serum lipid levels or percentages of prevalence, awareness, treatment, and control overall and in various population subgroups were estimated and compared by multiple linear regression or logistic regression. The multivariable logistic regression model was used to explore the associations between the socio-demographic factors and the prevalence, awareness, treatment and control of dyslipidemia.

**Results:**

The age- and sex-adjusted mean levels (95%*CI*) of total cholesterol (TC), triglyceride (TG), high-density lipoprotein cholesterol (HDL-C), and low-density lipoprotein cholesterol (LDL-C) were 4.76(4.75–4.77), 1.68(1.67–1.69),1.33(1.32–1.33), and 2.87 (2.86–2.88) mmol/L, respectively. Overall, the age-standardized prevalence of dyslipidemia was 32.21% (42.85% in men vs. 26.16% in women) in Chinese rural adults, with 5.11, 16.00, 19.27, and 4.76% for high TC, high TG, low HDL-C and high LDL-C, respectively. The age-standardized awareness, treatment and control of dyslipidemia were 15.07, 7.23, and 3.25%, respectively, which were higher in women than men, and increased steeply with age (*P*_*trend*_ < 0.05). The proportion of prevalence, awareness, treatment, control of dyslipidemia differed significantly among various subpopulations while the awareness, treatment, and control rates were universally low in all subgroups (< 54, 36, and 15%, respectively). Increasing age, men, unhealthy lifestyles, positive family history of dyslipidemia, abnormal weight, type 2 diabetes mellitus and hypertension were independent risk factors of dyslipidemia.

**Conclusion:**

Dyslipidemia was common with unacceptably low awareness, treatment and control rates in rural China. Therefore, effective strategies are necessary for improving the status of the prevention, diagnosis, treatment, control of dyslipidemia in Chinese rural residents.

**Trial registration:**

ChiCTR-OOC-15006699

## Background

Cardiovascular diseases (CVD) have become the leading cause of death with rapid economic development and adverse changes in lifestyle [1]. Although the recent prevalence of CVD has showed downward trend in many high-income countries, the global burden of CVD is still rising due to its increasing prevalence in lower- and middle-income countries where 80% of all global CVD-related deaths occur [2]. Large prospective studies have shown that dyslipidemia is a primary, widely established as an independent and modifiable major risk factor for cardiovascular disease, and successfully controlling lipid levels can decrease the risks of ischemic CVD [3–5]. Therefore, closely monitoring and controlling high risk factors of dyslipidemia are equally significant in achieving these goals.

Dyslipidemia has become an emerging epidemic in China with rapid economic development and adverse changes in lifestyle [6, 7]. Recent epidemiologic surveys in China has revealed that serum total cholesterol (TC) and low-density lipoprotein cholesterol (LDL-C) levels have increased than before, and the prevalence of dyslipidemia in the general population aged > 18 years old has reached up to 34.0% [6, 7]. Despite lower prevalence of dyslipidemia and levels of lipid in Chinese adults than those in many developed countries, the poor awareness, treatment, and control of dyslipidemia were common, especially in rural areas due to low economic status and incomplete health care systems [7, 8]. Thus, it would be extremely important for the further prevention and control of dyslipidemia and reducing the disease burden to know the data on the prevalence, awareness, treatment, control, as well as potential influencing factors of dyslipidemia.

Previous studies have concentrated on prevalence, awareness, treatment and control of dyslipidemia in China, however, the recent researches specialized in synthesizing to estimate the mean levels of serum lipids, prevalence, awareness, treatment, control across many heterogeneous subgroups, and risk factors in the limited medical service resources areas of rural China were scarce. Therefore, the aims of the present study are to estimate the mean levels of serum lipids, to provide data on the prevalence, awareness, treatment, and control of dyslipidemia overall and in various population subgroups, and to explore the relationships between potential risk factors and dyslipidemia in the Chinese rural population aged 18–79 years old.

## Methods

### Study subjects

The Henan Rural Cohort study was conducted in Yuzhou country, Suiping country, Xinxiang country, Tongxu county, and Yima county of Henan province in China from July 2015 to September 2017 [9]. The target population was residents aged 18–79 years who were permanently resident in the five rural areas. The cluster sampling design was used to select sample. In the first stage, 5 counties were selected from different geographical regions (south, central and north) in Henan Province in consideration of the adherence of the masses and local medical conditions. In the second stage, 1~ 3 typical rural districts in each county were selected by the local Centre for Disease Control and Prevention. In the final stage, all permanent residents who satisfied the inclusion criteria and signed informed consent in each sampled rural district were selected as the study sample. Overall, a total of 39,259 participants aged 18–79 years old in rural areas completed the survey, with a response rate of 93.7%. To estimate the mean levels of serum lipids, to provide data on the prevalence, awareness, treatment, and control of dyslipidemia overall and by age, sex, and other demographic characteristics, and to explore potential influencing factors in rural areas, 52 who lacked lipids data were excluded. Finally, 39,207 subjects were included for the present study. The Henan Rural Cohort study was approved by the Zhengzhou University Life Science Ethics Committee (Code: [2015] MEC (S128)). Written informed consent was obtained from each participants before data collection. The present study was conducted according to the 1975 Declaration of Helsinki.

### Measurements and definitions

Data were recorded by face-to-face interview. The trained research staff administered a standardized questionnaire to collect information on demographic characteristics, smoking, alcohol drinking, dietary habits, physical activities, personal history of disease, and family history of dyslipidemia. According to the smoking index (SI, SI = lifetime smoking intensity × duration of smoking) of the World Health Organization (WHO 1997), cigarette smoking status was categorized as never smoking, light smoking (0 < SI < 200), moderate smoking (200 ≤ SI < 400), and heavy smoking (SI ≥ 400). Alcohol drinking status was classified into never drinking, low risk drinking (0 < alcohol /day ≤25 g for men, 0 < alcohol/day ≤15 g for women), medium risk drinking (25 < alcohol/day l ≤ 60 g for men, 15 < alcohol/day ≤40 g for women), high risk drinking (alcohol/day > 60 g for men, alcohol/day > 40 g for women) in accordance with the daily intake amount of alcohol of Chinese Nutrition Society and WHO [10, 11]. Adequate vegetable and fruit intake was considered as a person who consumed an average of more than 500 g vegetable and fruit per day. High fat diet was defined as a person who took an average of more than 75 g meat of livestock and poultry per day in accordance with the dietary guidelines for Chinese residents [10]. Physical activity included three categories: low, moderate and high according to the international physical activity questionnaire (IPAQ 2001) [12].

Height and body weight were measured twice in light indoor clothing without shoes to the nearest 0.1 cm and 0.1 kg, respectively. Body mass index (BMI) was estimated as body weight (Kg) divided by height square (m^2^), and was classified into low weight (BMI < 18.5 kg/m^2^), normal weight (18.5 ≤ BMI < 24 kg/m^2^), overweight (24 ≤ BMI < 28 kg/m^2^), and obesity (BMI ≥ 28 kg/m^2^).

After at least 8 h of overnight fasting, venous blood specimen was collected in vacuum tubes without anticoagulation. Serum samples were separated from whole blood through centrifugation at for 10 min at 3000 rpm at room temperature, and then were sent to measure TC, triglycerides (TG), high-density lipoprotein cholesterol (HDL-C), LDL-C, and fasting blood glucose (FBG) by Roche Cobas C501 automatic biochemical analyzer. TC level was analyzed by cholesterol oxidase method, TG using enzymatic method, HDL-C and LDL-C by the direct method while FBG level was estimated by glucose oxidative method (GOD-PAP). Electronic sphygmomanometers (Omron HEM-7071A, Japan) were used to measure blood pressure on the right arm supported at heart level with sitting position for three times. The average of the three measurements was used for the statistic analysis. To have accurate readings, the participants were asked to have rest for at least 5 min, and have no tea and alcohol consumption, cigarette smoking, or excessive physical activity for at least 30 min or longer before the measurement.

According to the Chinese guidelines on prevention and treatment of dyslipidemia in adults [5], the cut-off values for high TC, high TG, low HDL-C and high LDL-C were 6.22 mmol/L (240 mg/dL), 2.26 mmol/L (200 mg/dL), 1.04 mmol/L (40 mg/dL), and 4.14 mmol/L (160 mg/dL), respectively. Dyslipidemia was defined as the presence of one or more abnormal serum lipid concentrations or use of anti-dyslipidemia medications in the past two weeks. Dyslipidemia awareness and treatment were defined, respectively, among dyslipidemia adults as a self-reported diagnosis of dyslipidemia, the application of lipid-lowering medications over the past 2 weeks. Dyslipidemia was considered to be controlled among participants with dyslipidemia if TC < 6.22 mmol/L (240 mg/dL), TG < 2.26 mmol/L (200 mg/dL), HDL-C > 1.04 mmol/L (40 mg/dL), and LDL-C < 4.14 mmol/L (160 mg/dL). Type 2 diabetes mellitus (T2DM) was diagnosed according to the American Diabetes Association (ADA) diagnostic criteria in subjects with FBG ≥7.0 mmol/L (126 mg/dL) or who had previously been diagnosed with diabetes by a physician [13]. Hypertension (HTN) was defined as the individual who had an average systolic blood pressure (SBP) ≥ 140 mmHg and/or an average diastolic blood pressure (DBP) ≥ 90 mmHg, or self-reported use of antihypertensive medications during the previous 2 weeks [14].

### Statistical analysis

All statistical analyses were performed using SAS9.1 software package (SAS Institute, USA). Continuous variables presented as mean ± standard deviation (SD) were compared using the *t*-test, while categorical variables presented as numbers and proportions were compared using chi-square test. The proportions of high TC, high TG, low HDL-C, and prevalence, awareness, treatment, control of dyslipidemia were standardized using the direct method according to the Chinese Population Census 2010 [15]. The age- and sex- adjusted means (95% confidence intervals, *CI*) of serum lipid levels or percentages of prevalence, awareness, treatment, and control of dyslipidemia by various characteristics were compared using multiple linear (for continuous outcomes) or logistic regression (for binary outcomes). The multivariable logistic regression model was used to calculate odds ratios (*OR*) and 95%*CI* between the potential influencing factors and the prevalence, awareness, treatment, and control of dyslipidemia in the full model. All tests were two-tailed and a *P* value of < 0.05 was considered as statistically significant.

## Results

Table 1 shows the demographic characteristics of the participants according to dyslipidemia status. Among the 39,207 participants aged 18–79 years old, a total of 14,744 were diagnosed with dyslipidemia (37.61%) with substantial imparity between men and women (40.20% vs. 35.92%), and the corresponding age-standardized prevalence of dyslipidemia for this population were 32.21% (42.85% in men and 26.16% in women). Compared with the subjects without dyslipidemia, the dyslipidemic subjects had the following characteristics: older age, men, higher percentage of tobacco smoking, drinking alcohol, positive family history of dyslipidemia, lack of physical activity (*P* < 0.05 for each). The mean levels of BMI, FBG, SBP, DBP, TC, TG and LDL-C were significantly higher in participants with dyslipidemia than those in the non-dyslipidemia participants, whereas the mean level of HDL-C was lower in dyslipidemic subjects (*P* < 0.001).

**Table 1:** Demographic characteristics of the participants

Variable	Total (*n* = 39,207)	With dyslipidemia (*n* = 14,744)	Without dyslipidemia (*n* = 24,463)	*χ* ^*2*^ */t*	*P*
Age (years), mean ± SD	55.60 ± 12.19	56.36 ± 11.42	55.14 ± 12.61	9.59	< 0.001
Men, n (%)	15,464 (39.44)	6216 (42.16)	9248 (37.80)	73.06	< 0.001
Marital status, n (%)				2.61	0.106
Married/cohabiting	35,197 (89.77)	13,283 (90.09)	21,914 (89.58)		
Widowed/single/divorced/separation	4010 (10.23)	1461 (9.91)	2549 (10.42)		
Education, n (%)				2.27	0.322
Primary school or lower education	17,554 (44.77)	6673 (45.26)	10,881 (61.99)		
Junior high school	15,621 (39.84)	5825 (39.51)	9796 (40.04)		
Senior higher education or above	6032 (15.39)	2246 (15.23)	3786 (15.48)		
Per capita monthly income, n (%)				0.079	0.961
≤500 RMB	13,989 (35.68)	5252 (35.62)	8737 (35.72)		
500 RMB ~	12,896 (32.89)	4846 (32.87)	8050 (32.91)		
≥1000 RMB	12,322 (31.43)	4646 (31.51)	7676 (31.38)		
Smoking, n (%)				66.54	< 0.001
Never	28,544 (72.80)	10,388 (70.46)	18,156 (74.22)		
Light	2200 (5.61)	910 (6.17)	1290 (5.27)		
Moderate	1770 (4.51)	733 (4.97)	1037 (4.24)		
Heavy	6693 (17.07)	2713 (18.40)	3980 (16.27)		
Drinking, n (%)				65.03	< 0.001
Never	30,392 (77.52)	11,155 (75.66)	19,237 (78.64)		
Low risk	5440 (13.88)	2144 (14.54)	3296 (13.47)		
Medium risk	1846 (4.71)	751 (5.09)	1095 (4.48)		
High risk	1529 (3.90)	694 (4.71)	835 (3.41)		
Adequate vegetable and fruit intake, n (%)	16,370 (41.75)	6195 (42.02)	10,175 (41.59)	0.70	0.404
High fat diet, n (%)	7472 (19.06)	2777 (18.83)	4695 (19.19)	0.76	0.383
Physical activity, n (%)				202.35	< 0.001
Low	12,691 (32.37)	5335 (36.18)	7356 (30.07)		
Moderate	14,791 (37.73)	5512 (37.38)	9297 (37.93)		
High	11,725 (29.91)	3897 (26.43)	7828 (32.00)		
Positive family history, n (%)	1393 (3.55)	616 (4.18)	777 (3.18)	26.94	< 0.001
BMI (kg/m^2^), mean ± SD	24.83 ± 3.57	26.06 ± 3.40	24.10 ± 3.46	54.60	< 0.001
FBG, mmol/L	5.54 ± 1.51	5.84 ± 1.79	5.37 ± 1.28	29.89	< 0.001
SBP, mmHg	125.95 ± 20.00	129.48 ± 19.75	123.83 ± 19.84	27.37	< 0.001
DBP, mmHg	77.70 ± 11.64	80.23 ± 11.44	76.17 ± 11.50	33.94	< 0.001
TC, mmol/L	4.76 ± 0.99	5.11 ± 1.23	4.55 ± 0.73	56.93	< 0.001
HDL-C, mmol/L	1.32 ± 0.33	1.12 ± 0.32	1.45 ± 0.28	− 103.84	< 0.001
LDL-C, mmol/L	2.87 ± 0.82	3.08 ± 1.04	2.74 ± 0.62	39.40	< 0.001
TG, mmol/L	1.68 ± 1.12	2.48 ± 1.43	1.20 ± 0.43	130.21	< 0.001

*BMI* body mass index, *FBG* fasting blood glucose, *SBP* systolic blood pressure, *DBP* diastolic blood pressure, *TC* total cholesterol, *HDL-C* high-density lipoprotein cholesterol, *LDL-C* low-density lipoprotein cholesterol, *TG* triglycerides, and *SD* standard deviation

Table 2 displays the mean (95% *CI*) of serum TC, TG, HDL-C, and LDL-C levels in Chinese rural population. The age- and sex-adjusted mean levels (95%*CI*) of TC, TG, HDL-C and LDL-C were 4.76(4.75–4.77), 1.68(1.67–1.69), 1.33(1.32–1.33), and 2.87(2.86–2.88) mmol/L, respectively. Overall, the age-adjusted mean levels of TC, TG, HDL-C, and LDL-C were significantly higher in women than in men. In general, TC, TG, and LDL-C levels were higher at age above 50 years while HDL-C levels were higher over the entire age range in women than men. The liner regression analysis showed that TC, HDL-C, and LDL-C levels increased with age in total population. In subgroup analysis, TC, TG, HDL-C, and LDL-C levels increased with age in women. For men, TC and TG levels decreased with age except the first age group, HDL-C levels increased with age, while LDL-C levels showed no obviously change trend.

**Table 2:** Mean (95% *CI*) of serum TC, TG, HDL-C, and LDL-C levels in Chinese rural population

Variable	TC (mmol/L)	TG (mmol/L)	HDL-C (mmol/L)	LDL-C (mmol/L)
Age-adjusted				
Total^a^	4.76 (4.75–4.77)	1.68 (1.67–1.69)	1.33 (1.32–1.33)	2.87 (2.86–2.88)
Men	4.63 (4.62–4.65)	1.66 (1.64–1.68)	1.26 (1.25–1.26)	2.81 (2.80–2.82)
Women	4.84 (4.83–4.86)	1.69 (1.67–1.70)	1.37 (1.37–1.37)	2.91 (2.90–2.92)
*P*	< 0.001	0.022	< 0.001	< 0.001
Total, age, y^a^				
18~	4.40 (4.37–4.43)	1.48 (1.44–1.51)	1.30 (1.29–1.31)	2.55 (2.52–2.57)
40~	4.61 (4.59–4.63)	1.71 (1.69–1.74)	1.29 (1.28–1.30)	2.74 (2.72–2.76)
50~	4.83 (4.81–4.85)	1.80 (1.78–1.82)	1.31 (1.30–1.31)	2.91 (2.90–2.93)
60~	4.86 (4.85–4.88)	1.67 (1.65–1.69)	1.35 (1.34–1.35)	2.96 (2.95–2.98)
70–79	4.91 (4.88–4.94)	1.54 (1.51–1.58)	1.38 (1.37–1.39)	3.01 (2.98–3.03)
*P*	< 0.001	< 0.001	< 0.001	< 0.001
*P*_*trend*_	< 0.001	0.828	< 0.001	< 0.001
Sex- and age-specific				
Men, age, y				
18~	4.61 (4.56–4.66)	1.89 (1.84–1.95)	1.16 (1.15–1.18)	2.75 (2.71–2.79)
40~	4.77 (4.73–4.80)	1.97 (1.93–2.02)	1.20 (1.19–1.21)	2.86 (2.83–2.89)
50~	4.67 (4.64–4.70)	1.77 (1.74–1.81)	1.24 (1.23–1.25)	2.83 (2.81–2.86)
60~	4.60 (4.57–4.63)	1.48 (1.45–1.51)	1.31 (1.30–1.31)	2.82 (2.79–2.84)
70–79	4.60 (4.56–4.64)	1.37 (1.32–1.42)	1.32 (1.31–1.34)	2.83 (2.80–2.86)
*P*	< 0.001	< 0.001	< 0.001	0.003
*P*_*trend*_	< 0.001	< 0.001	< 0.001	0.310
Women, age, y				
18~	4.28 (4.25–4.32)	1.24 (1.20–1.29)	1.38 (1.37–1.40)	2.43 (2.40–2.47)
40~	4.53 (4.50–4.56)	1.57 (1.53–1.60)	1.35 (1.34–1.36)	2.68 (2.66–2.70)
50~	4.93 (4.90–4.95)	1.82 (1.79–1.84)	1.35 (1.34–1.36)	2.96 (2.94–2.98)
60~	5.05 (5.03–5.07)	1.80 (1.78–1.83)	1.37 (1.36–1.38)	3.07 (3.05–3.09)
70–79	5.15 (5.11–5.19)	1.69 (1.65–1.73)	1.41 (1.40–1.43)	3.15 (3.12–3.18)
*P*	< 0.001	< 0.001	< 0.001	< 0.001
*P*_*trend*_	< 0.001	< 0.001	< 0.001	< 0.001

*CI* confidence interval, *TC* total cholesterol, *TG* triglycerides, *HDL-C* high-density lipoprotein cholesterol, *LDL-C* low-density lipoprotein cholesterol

^a^In addition adjusted for sex

Table 3 describes the age-standardized proportion of serum high TC, high TG, low HDL-C, and high LDL-C in Chinese rural population. In the 39,207 participants, the number of patients with high TC, high TG, low HDL-C and high LDL-C were 2911, 7340, 7593, and 2629, respectively. The corresponding age-standardized prevalence were 5.11, 16.00, 19.27, and 4.76% in the rural population: 5.27, 29.51, 5.58, 21.11% in men and 5.26, 13.12, 4.47, 13.33% in women, respectively. The age-standardized prevalence of high TC and high LDL-C increased continuously over the entire age range in total population and women, whereas the age-standardized prevalence of high TG increased with age until 50 years in men and 60 years in women, and then decreased. There was a tendency of decrease in men in the age-adjusted proportion of low HDL-C.

**Table 3:** Age-standardized proportion of serum high TC, high TG, low HDL-C, and high LDL-C in Chinese rural population

Variable	High TC, n(%)	High TG, n(%)	Low HDL-C, n(%)	High LDL-C, n(%)
Age-standardized				
Total	2911 (5.11)	7340 (16.00)	7593 (19.27)	2629 (4.76)
Men	883 (5.27)	2820 (21.11)	3990 (29.51)	892 (5.58)
Women	2028 (5.26)	4520 (13.33)	3603 (13.12)	1737 (4.47)
*P*	< 0.001	0.072	< 0.001	< 0.001
Total, age, y				
18~	129 (2.88)	581 (11.76)	838 (18.20)	125 (2.98)
40~	380 (4.96)	1465 (20.04)	1607 (22.13)	338 (4.41)
50~	804 (7.68)	2431 (22.82)	2212 (20.62)	705 (6.74)
60~	1071 (8.68)	2210 (18.00)	2198 (17.95)	993 (8.12)
70–79	527 (11.37)	653 (13.22)	738 (15.29)	468 (9.97)
*P*	< 0.001	< 0.001	< 0.001	< 0.001
*P*_*trend*_	< 0.001	0.001	< 0.001	< 0.001
Men, age, y				
18~	77 (4.06)	372 (20.45)	527 (31.64)	88 (5.13)
35~	203 (7.53)	730 (28.69)	853 (32.35)	175 (6.45)
45~	221 (5.72)	845 (21.34)	1080 (27.77)	206 (5.48)
55~	253 (4.83)	655 (12.66)	1120 (21.47)	282 (5.43)
65–74	129 (5.98)	218 (9.53)	410 (18.35)	141 (6.47)
*P*	< 0.001	< 0.001	< 0.001	0.095
*P*_*trend*_	0.031	< 0.001	< 0.001	0.624
Women, age, y				
18~	52 (2.32)	209 (6.87)	311 (10.09)	37 (1.86)
35~	177 (3.46)	735 (15.14)	754 (16.24)	163 (3.25)
45~	583 (8.89)	1586 (23.85)	1132 (16.57)	499 (7.49)
55~	818 (11.53)	1555 (21.98)	1078 (15.39)	711 (10.11)
65–74	398 (15.99)	435(16.36)	328 (12.71)	327 (12.95)
*P*	< 0.001	< 0.001	< 0.001	< 0.001
*P*_*trend*_	< 0.001	< 0.001	0.855	< 0.001

*TC* total cholesterol, *TG* triglycerides, *HDL-C* high-density lipoprotein cholesterol, *LDL-C* low-density lipoprotein cholesterol, Age-standardized according to the Chinese Population Census 2010 by the direct method

Table 4 presents the prevalence, awareness, treatment, and control of dyslipidemia among different characteristics. In the 14,744 dyslipidemic participants, 3420 were aware of the diagnosis (23.20%), 1857 were taking medication (12.96%), and 886 had their blood pressure controlled (6.01%). The corresponding age-standardized awareness, treatment and control of dyslipidemia were 15.07, 7.23, and 3.25%, respectively. The proportion of prevalence, awareness, treatment, and control of dyslipidemia varied significantly across subpopulations. Meanwhile, the awareness, treatment, and control rates were universally low in all subgroups (< 54, 36, and 15%, respectively). Those who were older, man, married/cohabiting, smoking, drinking, with inadequate physical activity, positive family history of dyslipidemia, abnormal weight, T2DM and HTN were susceptive to dyslipidemia. Lower likelihoods of awareness and treatment were associated with younger, man, smoker, drinker, with adequate physical activity, and an absence of previous T2DM, HTN, obesity, or positive family history of dyslipidemia (all *P* < 0·05).

**Table 4:** Adjusted proportion (95% *CI*) of prevalence, awareness, treatment, and control of dyslipidemia among different characteristics (*N* = 39,207)

Variable	Prevalence	Dyslipidemia		
		Awareness	Treatment	Control
Age (years)^a^				
18~	29.09 (27.71–30.51)	5.40 (4.23–6.86)	1.30 (0.79–2.15)	0.09 (0.01–0.61)
40~	36.56 (35.46–37.69)	13.37 (12.12–14.72)	4.93 (4.17–5.82)	1.65 (1.23–2.21)
50~	41.44 (40.50–42.38)	25.77 (23.80–26.39)	11.97 (11.04–12.97)	5.32 (4.69–6.02)
60~	39.30 (38.44–40.18)	27.77 (26.50–29.07)	16.77 (15.73–17.87)	8.57 (7.79–9.41)
70~ 79	37.00 (35.63–38.38)	27.20 (25.17–29.33)	18.46 (16.72–20.34)	9.30 (8.03–10.74)
*P*	< 0.001	< 0.001	< 0.001	< 0.001
*P*_*trend*_	< 0.001	< 0.001	< 0.001	< 0.001
Gender^b^				
Women	36.01 (35.40–36.62)	25.03 (24.10–25.98)	12.72 (12.01–13.46)	5.84 (5.34–6.37)
Men	39.98 (39.21–40.76)	18.03 (17.82–19.79)	9.46 (8.75–10.23)	4.15 (3.68–4.68)
*P*	< 0.001	< 0.001	< 0.001	< 0.001
Marital status^c^				
Married/cohabiting	38.24 (37.72–38.76)	21.76 (21.04–22.50)	11.02 (10.46–11.60)	4.87 (4.50–5.28)
Widowed/single/divorced/separation	35.62 (34.13–37.14)	21.58 (19.58–23.73)	10.64 (9.28–12.18)	5.51 (4.56–6.65)
*P*	0.001	0.873	0.620	0.200
Education^c^				
Primary school or lower education	37.61 (36.81–38.41)	21.39 (20.30–22.51)	11.04 (10.22–11.91)	5.12 (4.56–5.75)
Junior high school	38.05 (37.28–38.83)	21.82 (20.74–22.94)	10.92 (10.11–11.78)	4.74 (4.21–5.34)
Senior higher education or above	38.74 (37.45–40.40)	22.52 (20.67–24.47)	11.02 (9.66–12.56)	4.91 (4.01–6.01)
*P*	0.133	< 0.001	< 0.001	< 0.001
*P*_trend_	0.133	< 0.001	< 0.001	< 0.001
Per capita monthly income				
≤ 500 RMB	37.17 (36.35–37.99)	22.04 (20.89–23.19)	11.05 (10.22–11.94)	4.61 (4.09–5.20)
500 RMB ~	38.14 (37.29–38.99)	20.27 (19.15–21.45)	10.28 (9.45–11.17)	4.60 (4.05–5.22)
≥ 1000 RMB	38.72 (37.84–39.60)	22.96 (21.74–24.24)	11.65 (10.73–12.64)	5.61 (4.97–6.33)
*P*	0.961	< 0.001	< 0.001	0.042
*P*_trend_	0.790	< 0.001	< 0.001	0.078
Smoking^c^				
Never	37.28 (36.54–38.02)	22.10 (21.04–23.19)	11.43 (10.63–12.29)	4.98 (4.44–5.58)
Light	40.90 (38.73–43.10)	20.53 (17.60–23.82)	9.84 (7.77–12.38)	4.59 (3.21–6.52)
Moderate	40.39 (38.02–42.81)	21.68 (18.44–25.32)	11.64 (9.21–14.60)	5.19 (3.61–7.41)
Heavy	38.50 (37.13–39.89)	21.12 (19.29–23.08)	9.91 (8.66–11.33)	4.81 (3.94–5.88)
*P*	< 0.001	< 0.001	< 0.001	< 0.001
*P*_trend_	< 0.001	< 0.001	< 0.001	< 0.001
Drinking^c^				
Never	37.31 (36.68–37.94)	21.05 (20.16–21.97)	10.77 (10.09–11.48)	4.62 (4.17–5.11)
Low risk	38.53 (37.16–39.93)	22.06 (20.08–24.18)	11.17 (9.70–12.81)	6.04 (4.95–7.35)
Medium risk	39.62 (37.34–41.93)	24.55 (21.22–28.21)	11.09 (8.78–13.92)	5.49 (3.89–7.71)
High risk	43.94 (41.41–46.51)	26.86 (23.34–30.71)	13.19 (10.61–16.28)	5.17 (3.60–7.38)
*P*	< 0.001	< 0.001	< 0.001	0.002
*P*_trend_	< 0.001	< 0.001	< 0.001	< 0.001
Adequate vegetable and fruit intake^c^	38.33 (37.58–39.08)	25.72 (24.62–26.85)	12.19 (11.39–13.05)	5.35 (4.81–5.94)
*P*	0.220	< 0.001	< 0.001	0.028
High fat diet^c^	37.78 (36.67–38.90)	21.95 (20.36–23.62)	9.50 (8.41–10.72)	3.94 (3.25–4.78)
*P*	0.698	0.784	0.008	0.011
Physical activity^c^				
Low	42.07 (41.20–42.98)	19.31 (18.26–20.41)	10.27 (9.48–11.13)	3.94 (3.46–4.48)
Moderate	38.30 (37.49–39.12)	23.43 (22.29–24.61)	11.65 (10.80–12.55)	5.25 (4.69–5.88)
High	33.28 (32.43–34.14)	22.74 (21.43–24.10)	11.03 (10.08–12.05)	5.74 (5.06–6.50)
*P*	< 0.001	< 0.001	0.201	0.042
*P*_trend_	< 0.001	0.019	0.345	0.021
Family history^c^	54.33 (52.07–56.57)	53.88 (49.76–57.95)	35.18 (31.13–39.46)	15.02 (12.13–18.46)
*P*	< 0.001	< 0.001	< 0.001	< 0.001
BMI^c^				
Underweight	10.89 (9.07–13.02)	15.55 (10.25–22.89)	8.34 (4.88–13.90)	6.18 (3.36–11.08)
Normal	25.51 (24.83–26.20)	17.41 (16.26–18.61)	9.16 (8.32–10.07)	5.44 (4.79–6.17)
Overweight	44.40 (43.61–45.20)	21.74 (20.75–22.76)	10.57 (9.85–11.33)	4.55 (4.07–5.07)
Obesity	56.41 (55.23–57.59)	26.43 (25.02–27.89)	13.64 (12.57–14.79)	5.02 (4.38–5.76)
*P*	< 0.001	< 0.001	< 0.001	< 0.001
*P*_*trend*_	< 0.001	< 0.001	< 0.001	0.002
T2DM^c^	58.39 (56.77–59.98)	30.35 (28.44–32.34)	16.82 (15.33–18.42)	6.13 (5.26–7.14)
*P*	< 0.001	< 0.001	< 0.001	0.002
HTN^c^	48.62 (47.72–49.52)	28.04 (26.88–29.22)	16.57 (15.60–17.58)	6.72 (6.09–7.42)
*P*	< 0.001	< 0.001	< 0.001	< 0.001

*CI* confidence interval, *BMI* body mass index, *T2DM* type 2 diabetes mellitus, *HTN* hypertension

*P* values from chi-square test. *P*_*trend*_ values from the trend chi-square test

^a^adjusted for sex; ^b^adjusted for age; ^c^adjusted for sex and age

**Fig. 1:**
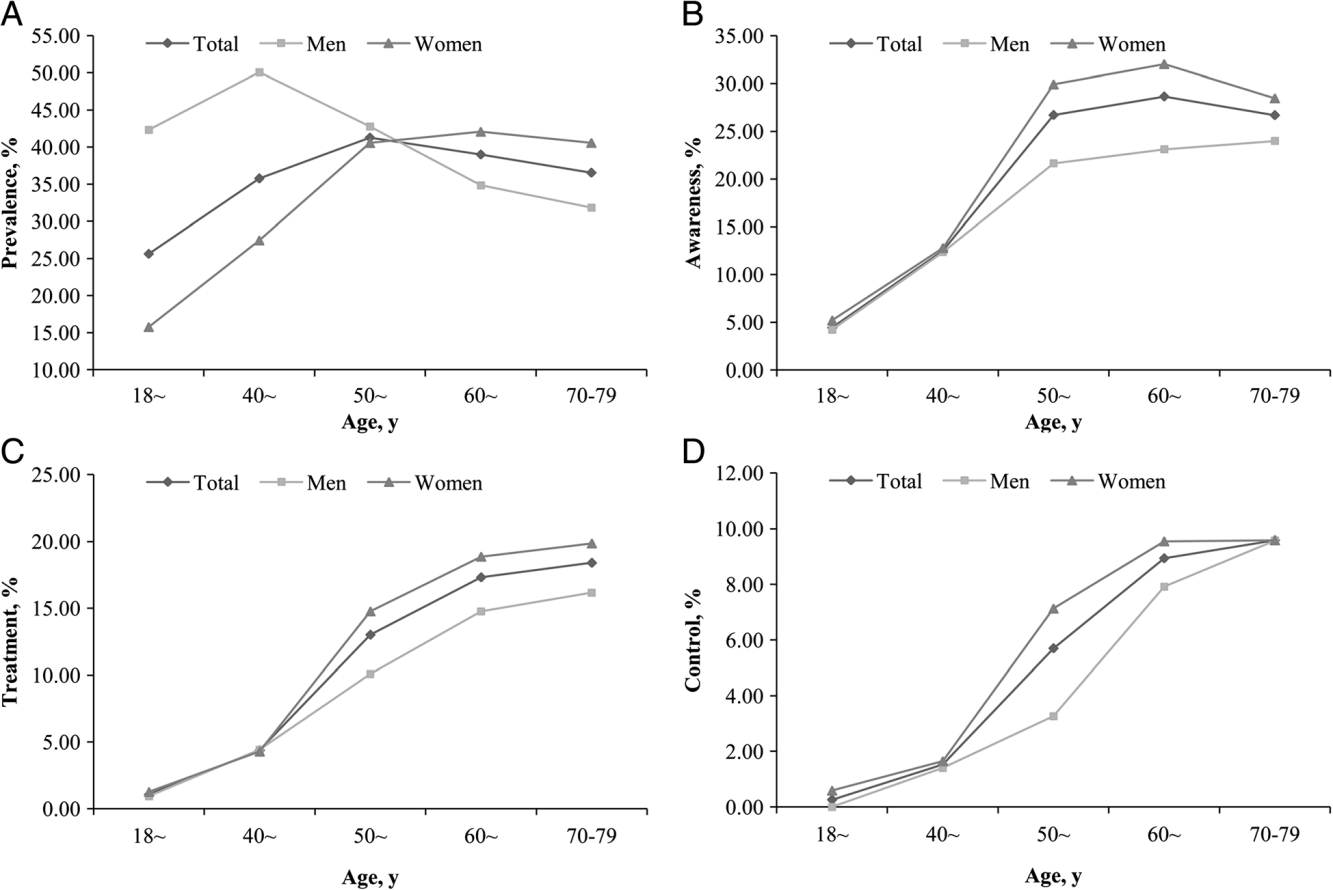
Changes in the age-standardized prevalence, awareness, treatment, and control of dyslipidemia with aging in different sexes. **a** is for prevalence, **b** is for awareness, **c** is for treatment, and **d** is for control

Figure 1 describes changes in the age-standardized prevalence, awareness, treatment, and control of dyslipidemia with aging in different sexes. The age-standardized awareness, treatment and control of dyslipidemia in men and women were 11.32% vs. 19.12, 4.94% vs. 9.58, 1.87% vs.4.59%, respectively. Men ages 40 to 49 years had the highest prevalence, while. Women generally had significantly increase in the prevalence of dyslipidemia, with peak range between 60 and 69 years. Women had a higher prevalence of dyslipidemia than men above the age of 60, but lower prevalence when they were in premenopausal and perimenopausal period (Fig. 1a). The age-standardized awareness, treatment, and control of dyslipidemia increased continuously over the entire age range in both sexes (*P*_*trend*_ < 0.05), and were higher in women than those in men on the whole (Fig. 1b, c and d).

Table 5 displays the results of multivariate logistic regression analysis for potential influencing factors associated with prevalence, awareness, treatment, and control of dyslipidemia. Increasing age, men, cigarette smoking, adequate vegetable and fruit intake, inadequate physical activity, positive family history of dyslipidemia, abnormal weight, T2DM and HTN were independent risk factors of dyslipidemia. Those who were older, T2DM, HTN, taking adequate vegetable and fruit intake, high physical activity, and had positive family history of dyslipidemia were more likely to be aware of their dyslipidemia conditions, to be seeking treatment, and to have their serum lipid level controlled to standard level while men and high fat diet had smaller proportions of awareness, treatment, and control of dyslipidemia. Excess BMI was positively associated with awareness, treatment, and was negatively related to the control rate of dyslipidemia.

**Table 5:** Associations between potential risk factors and dyslipideima

Variable	Prevalence OR(95% CI)	Dyslipidemia		
		Awareness OR(95% CI)	Treatment OR(95% CI)	Control OR(95% CI)
Age				
18~	1.00	1.00	1.00	/
40~	1.22 (1.12–1.34)	2.85 (2.12–3.82)	3.74 (2.16–6.48)	1.00
50~	1.41 (1.29–1.54)	6.28 (4.73–8.32)	9.69 (5.71–16.45)	3.16 (2.28–4.40)
60~	1.30 (1.19–1.42)	7.23 (5.43–9.65)	13.58 (7.96–23.147)	4.98 (3.58–6.91)
70~ 79	1.17 (1.05–1.31)	7.21 (5.31–9.78)	14.85 (8.60–25.63)	5.23 (3.64–7.52)
*P*_trend_	0.001	< 0.001	< 0.001	< 0.001
Gender				
Women	1.00	1.00	1.00	1.00
Men	1.20 (1.12–1.29)	0.75 (0.65–0.86)	0.86 (0.72–1.02)	0.75 (0.59–0.97)
Marital status				
Married/cohabiting	1.00	1.00	1.00	1.00
Widowed/single/divorced/separation	1.01 (0.94–1.09)	1.13 (0.99–1.29)	1.07 (0.91–1.25)	1.27 (1.03–1.55)
Education				
Primary school or lower education	1.00	1.00	1.00	1.00
Junior high school	0.99 (0.94–1.04)	0.95 (0.87–1.05)	0.94 (0.83–1.07)	0.88 (0.74–1.04)
Senior higher education or above	0.99 (0.93–1.07)	0.97 (0.84–1.11)	0.91 (0.76–1.10)	0.90 (0.70–1.17)
*P*_*trend*_	0.815	0.797	0.481	< 0.001
Per capita monthly income				
≤ 500 RMB	1.00	1.00	1.00	1.00
500 RMB ~	0.98 (0.93–1.03)	0.87 (0.79–0.96)	0.91 (0.80–1.03)	0.99 (0.84–1.17)
≥ 1000 RMB	1.01 (0.96–1.07)	0.99 (0.90–1.10)	1.04 (0.92–1.19)	1.23 (1.03–1.47)
*P*_*trend*_	0.650	0.876	0.420	0.208
Smoking				
Never	1.00	1.00	1.00	1.00
Light	1.27 (1.14–1.41)	0.99 (0.79–1.24)	0.91 (0.68–1.23)	0.94 (0.62–1.44)
Moderate	1.24 (1.10–1.39)	1.08 (0.85–1.37)	1.13 (0.84–1.53)	1.08 (0.70–1.66)
Heavy	1.17 (1.07–1.27)	0.95 (0.81–1.10)	0.90 (0.74–1.11)	0.96 (0.72–1.28)
*P*_*trend*_	0.001	0.795	0.838	0.310
Drinking				
Never	1.00	1.00	1.00	1.00
Low risk	0.97 (0.90–1.04)	1.03 (0.90–1.20)	1.01 (0.83–1.22)	1.30 (1.01–1.69)
Medium risk	0.92 (0.82–1.02)	1.16 (0.94–1.44)	1.00 (0.75–1.34)	1.18 (0.79–1.78)
High risk	1.06 (0.94–1.19)	1.20 (0.96–1.49)	1.10 (0.83–1.46)	1.07 (0.71–1.63)
*P*_*trend*_	0.881	0.057	0.639	0.943
Adequate vegetable and fruit intake	1.12 (1.07–1.17)	1.53 (1.41–1.66)	1.30 (1.17–1.45)	1.16 (1.00–1.34)
High fat diet	0.95 (0.89–1.00)	0.91 (0.81–1.02)	0.76 (0.65–0.89)	0.71 (0.57–0.89)
Physical activity				
Low	1.00	1.00	1.00	1.00
Moderate	0.89 (0.84–0.93)	1.26 (1.14–1.38)	1.17 (1.03–1.32)	1.31 (1.10–1.55)
High	0.70 (0.66–0.74)	1.21(1.09–1.34)	1.08 (0.94–1.24)	1.40 (1.17–1.68)
*P*_*trend*_	< 0.001	< 0.001	0.030	< 0.001
Positive family history	1.41 (1.25–1.58)	4.95 (4.11–5.96)	5.12 (4.16–6.32)	3.78 (2.88–4.96)
BMI				
Normal	1.00	1.00	1.00	1.00
Underweight	0.39 (0.32–0.48)	1.12 (0.68–1.82)	1.16 (0.64–2.10)	1.41 (0.74–2.71)
Overweight	2.15 (2.05–2.26)	1.20 (1.08–1.32)	1.01 (0.89–1.15)	0.74 (0.63–0.87)
Obesity	3.18 (2.99–3.38)	1.42 (1.26–1.59)	1.17 (1.01–1.35)	0.75 (0.62–0.91)
*P*_*trend*_	< 0.001	< 0.001	0.051	< 0.001
T2DM	2.06 (1.91–2.21)	1.61 (1.45–1.79)	1.67 (1.48–1.89)	1.27 (1.06–1.51)
HTN	1.50 (1.43–1.58)	1.78 (1.64–1.94)	2.48 (2.22–2.77)	1.96 (1.69–2.28)

*BMI* body mass index, *T2DM* type 2 diabetes mellitus, *HTN* hypertension, *OR* odds ratio, *CI* confidence interval

## Discussion

The present large survey specialized in Chinese rural population provides important new evidence about the current burden of dyslipidemia in China, particularly attributed to its undiagnosed, without treatment and uncontrolled. Overall, the mean levels of TC, TG and LDL-C were much higher, whereas HDL-C levels were lower than the previous studies in the general rural Chinese adult population [14]. Furthermore, this study indicated that one third of the rural adult population in China had dyslipidemia (The proportions of high TC, high TG, low HDL-C, and high LDL-C were 5.11, 16.00, 19.27, and 4.76%, respectively). High TG and low HDL-C have become two major types of dyslipidemia in Chinese rural adults. The prevalence increased steeply with age, and varied substantially by sex. Among those with dyslipidemia, the proportions of patients who were aware, treated, and controlled were very low (15.07, 7.23, and 3.25%, respectively), especially in men. The proportion of prevalence, awareness, treatment, and control of dyslipidemia varied significantly across subpopulations. Meanwhile, the awareness, treatment, and control rates were universally low in all subgroups. Increasing age, men, adequate vegetable and fruit intake, inadequate physical activity, positive family history of dyslipidemia, increased BMI, T2DM, and HTN were associated risk factors for dyslipidemia.

Evidences from previous epidemiological studies showed that lipid levels changed dramatically in Chinese populations [7, 16, 17]. Similar results were founded in our study. The mean levels (95%*CI*) of TC, TG, HDL-C, and LDL-C were 4.76(4.75–4.77), 1.68(1.67–1.69), 1.33(1.32–1.33), and 2.87 (2.86–2.88) mmol/L, respectively. Despite of adjusting for sex and age, the mean levels of TC, TG, HDL-C, and LDL-C showed 2.15% (or 0.10 mmol/L), 9.80% (or 0.15 mmol/L), 2.31% (or 0.03 mmol/L), and 11.24% (or 0.29 mmol/L) compared with the national data in the study of Yang et al. [7] conducted in 2007–2008, respectively. Due to changes in lipid levels, the prevalence of dyslipidemia has been increasing during the past decade. The overall prevalence of dyslipidemia, at 32.21% in the current large sample of Chinese rural population, was obviously higher than the national rural data (26.3%) in the study of Pan et al. conducted in 2009–2010 [6]. High TG and low HDL-C have become two major subtypes of dyslipidemia in rural China [6, 17, 18]. The results in our study are consistent with the finding.

Recent studies found that high TG, low HDL-C, high TC/HDL-C and high LDL-C were associated with increased incidence of nonfatal and fatal ischemic stroke as well as all cerebrovascular events [4, 19, 20]. Therefore, effective preventive measures are needed to maintain lipid levels close to normal and decrease the incidence of dyslipidemia as well as related complications. Over the past decade, the great improvements in lipid levels attainment have been made in China, however, the rates of awareness, treatment, and control were low in the Chinese rural population [6]. In our study, among participants with dyslipidemia, 15.07% were aware of the diagnosis, 7.23% were receiving treatment, and only 3.25% had lipid levels controlled, which were obviously lower than those in developed countries and cities in China [18, 21]. What’s more, the subgroup analysis showed that the awareness, treatment, and control rates were universally low in all subgroups. Thus, more attention given to screening and health education should be paid to improve the status of the prevention, diagnosis, treatment, control of dyslipidemia and reduce the disease burden, especially in rural areas with limited health care resources.

Previous studies have declared that the prevalence of dyslipidemia was higher in men than women, whereas the rates of awareness, treatment, and control were lower in men than women. Similar results were found in our study. These data implied that the dyslipidemia management guidelines in our country should post men as a priority management group. But postmenopausal women had high levels of lipid and prevalence of dyslipidemia [6, 18], which was consistent with the findings of our survey. So postmenopausal women also should be the other key objects of prevention and treatment. Previous epidemiological studies have found that unhealthy lifestyle (smoking and inadequate physical activity), positive family history of dyslipidemia, and chronic non-communicable diseases (increased BMI, T2DM, and HTN) were associated with the prevalence and management rates of dyslipidemia [6, 22, 23]. Our results were in accordance with the findings. In addition, lifestyle changes were effective in controlling serum blood lipids [22, 24]. Therefore, there is an increased need for closely monitoring and controlling high risk factors of dyslipidemia including older, men, postmenopausal women, unhealthy lifestyle peoples and patients with chronic non-communicable diseases in Chinese rural areas. The effects of fruit and vegetable consumption on plasma lipid levels have not yet been thoroughly explored [25]. The positive association was found between adequate vegetable, fruit intake and dyslipidemia in the current study. Some subjects might have changed their lifestyle after knowing their lipid levels, which might have been responsible for the positive association. The prospective cohort study is needed to explore the association between adequate vegetable and fruit intake and lipid levels.

Previous studies have declared that increasing age, women, overweight, obesity, T2DM, HTN, and had positive family history of dyslipidemia were positively associated with awareness of dyslipidemia [22]. Similar results were found in the current study. These might be explained by that when people were older, with positive family history of dyslipidemia, and chronic non-communicable diseases, they concerned on their health conditions and were more likely to be aware of their dyslipidemia conditions. Contrary to other findings [22], the results of our study revealed that those who took adequate vegetable and fruit intake, high physical activity were more likely to be aware of their dyslipidemia conditions. The potential reason for the phenomenon might be some subjects might have changed dietary habits and exercise awareness after knowing their lipid levels. The prospective cohort study is needed to explore the causal associations.

The current study synthesized the epidemiologic characteristics and influencing factors of dyslipidemia based on a relatively large sample size of rural population in China. The standardized survey tools, training and field implementation, and adjusting for a wide range of potential confounders guarantee the reliability of the analysis. However, several limitations should also be addressed. Firstly, these findings were derived from a cross-sectional study not prospective cohort design, no causal relationships could be precisely delineated for the properties of the cross-sectional study. Secondly, a comprehensive range of potential confounders was adjusted in our analyses, but residual confounding or confounding by unmeasured or unknown factors might still exist. Furthermore, some subjects might have changed their lifestyle after knowing their lipid levels, which might lead to the selective bias. Finally, the results were based on a geographical region design, which might limit the representation. Therefore, incorporating multi-center data should be considered when the Henan Rural Cohort study is further established and modified. Although the present study has these limitations, the results based on a relatively large rural epidemiological study could reflect the prevalence of dyslipidemia in Chinese rural areas in some degree.

## Conclusion

The mean levels of TC, TG, HDL-C and LDL-C changed dramatically compared with the previous reports, and high TG and low HDL-C have become two major subtypes of dyslipidemia in rural China. Dyslipidemia prevalence was high, with unacceptably low awareness, treatment and control rates in rural China. Therefore, more attention should be paid to the prevention and control high risk factors in the populations including older, men, postmenopausal women, unhealthy lifestyle peoples and patients with chronic non-communicable diseases in Chinese rural areas. Effective measures are urgently needed to improve the status of the prevention, diagnosis, treatment, control of dyslipidemia and reduce the disease burden in Chinese rural residents.

## Abbreviations

BMI: Body mass index
CI: Confidence interval
CVD: Cardiovascular diseases
DBP: Diastolic blood pressure
FBG: Fasting blood glucose
HDL-C: High-density lipoprotein cholesterol
HTN: Hypertension
LDL-C: low-density lipoprotein cholesterol
OR: Odds ratio
SBP: Systolic blood pressure
SD: Standard deviation
T2DM: type 2 diabetes mellitus
TC: Total cholesterol
TG: Triglycerides

## References

[1] Roth GA, Johnson C, Abajobir A, Abd-Allah F, Abera SF, Abyu G (2017). Global, regional, and National Burden of cardiovascular diseases for 10 causes, 1990 to 2015. J Am Coll Cardiol.

[2] Farzadfar F, Finucane MM, Danaei G, Pelizzari PM, Cowan MJ, Paciorek CJ (2011). National, regional, and global trends in serum total cholesterol since 1980: systematic analysis of health examination surveys and epidemiological studies with 321 country-years and 3·0 million participants. Lancet.

[3] Lee JS, Chang PY, Zhang Y, Kizer JR, Best LG, Howard BV (2017). Triglyceride and HDL-C dyslipidemia and risks of coronary heart disease and ischemic stroke by glycemic dysregulation status: the strong heart study. Diabetes Care.

[4] Pikula A, Beiser AS, Wang J, Himali JJ, Kelly-Hayes M, Kase CS (2015). Lipid and lipoprotein measurements and the risk of ischemic vascular events: Framingham study. Neurology.

[5] Joint Committee for Developing Chinese Guidelines on Prevention and Treatment of Dyslipidemia in Adults. Chinese guidelines on prevention and treatment of dyslipidemia in adults. Zhonghua Xin Xue Guan Bing Za Zhi. 2007; 35: 390–419. 17711682

[6] Pan L, Yang Z, Wu Y, Yin RX, Liao Y, Wang J (2016). The prevalence, awareness, treatment and control of dyslipidemia among adults in China. Atherosclerosis.

[7] Yang W, Xiao J, Yang Z, Ji L, Jia W, Weng J (2012). Serum lipids and lipoproteins in Chinese men and women. Circulation.

[8] Zhao WH, Zhang J, You Y, Man QQ, Li H, Wang CR (2005). Epidemiologic characteristics of dyslipidemia in people aged 18 years and over in China. Zhonghua yu fang yi xue za zhi.

[9] Liu X, Li Y, Li L, Zhang L, Ren Y, Zhou H (2016). Prevalence, awareness, treatment, control of type 2 diabetes mellitus and risk factors in Chinese rural population: the RuralDiab study. Sci Rep.

[10] Chinese Nutrition Society (2011). The dietary guidelines for Chinese residents.

[11] World Health Organization (2000). International guide for monitoring alcohol consumption and related harm.

[12] Craig CL, Marshall AL, Sjöström M, Bauman AE, Booth ML, Ainsworth BE, et al. International physical activity questionnaire: 12-country reliability and validity. Med Sci Sports Exerc. 2003;35:1381–95. 10.1249/01.MSS.0000078924.61453.FB 12900694

[13] American Diabetes Association (2009). Diagnosis and classification of diabetes mellitus. Diabetes Care.

[14] Writing group of 2010 Chinese guidelines for the management of hypertension. 2010 Chinese guidelines for the management of hypertension. Chin J Hypertens. 2011; 19: 701–743.

[15] National Bureau of Statistics of the People’s Republic of China. China statistic press; 2010. http://www.stats.gov.cn/tjsj/pcsj/rkpc/6rp/html/A0301a.htm. Accessed 25 Apr 2016.

[16] He J, Gu D, Reynolds K, Wu X, Muntner P, Zhao J (2004). Serum total and lipoprotein cholesterol levels and awareness, treatment, and control of hypercholesterolemia in China. Circulation.

[17] Li LM, Rao KQ, Kong LZ, Yao CH, Xiang HD, Zhai FY (2005). A description on the Chinese national nutrition and health survey in 2002. Chin J Epidemiol.

[18] Li JH, Wang LM, Li YC, Bi YF, Jiang Y, Mi SQ (2012). Epidemiologic characteristics of dyslipidemia in Chinese adults in 2010. Chin J Prev Med.

[19] Holme I, Aastveit AH, Hammar N, Jungner I, Walldius G (2009). Relationships between lipoprotein components and risk of ischaemic and haemorrhagic stroke in the apolipoprotein mortality RISk study (AMORIS). J Intern Med.

[20] White J, Swerdlow DI, Preiss D, Fairhurst-Hunter Z, Keating BJ, Asselbergs FW (2016). Association of Lipid Fractions with Risks for coronary artery disease and diabetes. JAMA Cardiol.

[21] Benjamin EJ, Blaha MJ, Chiuve SE, Cushman M, Das SR, Deo R (2017). Heart disease and stroke Statistics-2017 update: a report from the American Heart Association. Circulation.

[22] He H, Yu YQ, Li Y, Kou CG, Li B, Tao YC (2014). Dyslipidemia awareness, treatment, control and influence factors among adults in the Jilin province in China: a cross-sectional study. Lipids Health Dis.

[23] Zhang J, Wang H, Yu M, Hu RY, Su DT, Zhao M (2015). Prevalence of dyslipidemia among non—overweight adults and related factors in Zhejiang. Chin J Epidemiol..

[24] Cai L, Zhang L, Liu A, Li S, Wang P (2012). Prevalence, awareness, treatment, and control of dyslipidemia among adults in Beijing, China. J Atheroscler Thromb.

[25] Dauchet L, Amouyel P, Dallongeville J (2009). Fruits, vegetables and coronary heart disease. Nat Rev Cardiol.

